# Digital Peer-Supported App Intervention to Promote Physical Activity Among Community-Dwelling Older Adults: Nonrandomized Controlled Trial

**DOI:** 10.2196/56184

**Published:** 2024-05-30

**Authors:** Kento Tabira, Yuko Oguma, Shota Yoshihara, Megumi Shibuya, Manabu Nakamura, Natsue Doihara, Akihiro Hirata, Tomoki Manabe

**Affiliations:** 1 Graduate School of Health Management, Keio University Kanagawa Japan; 2 Sports Medicine Research Center, Keio University Kanagawa Japan; 3 Department of Rehabilitation Sciences, Graduate School of Medical Sciences, Kitasato University Kanagawa Japan; 4 A10 Lab Inc Tokyo Japan; 5 Japan Society for the Promotion of Science Tokyo Japan

**Keywords:** physical activity, physical function, gerontology, geriatric, geriatrics, older adult, older adults, elder, elderly, older person, older people, ageing, aging, aged, digital peer support app, mHealth, mobile health, app, apps, application, applications, eHealth, peer support, exercise, mobile phone

## Abstract

**Background:**

The use of mobile apps has promoted physical activity levels. Recently, with an increasing number of older adults accessing the internet, app-based interventions may be feasible in older populations. Peer support–based interventions have become a common method for promoting health-related behavior change. To our knowledge, the feasibility of using digital peer support apps (DPSAs) to increase physical activity among older adults and its impact on physical activity and physical function have not been investigated.

**Objective:**

This study aims to assess the feasibility of using DPSAs in older adults and to assess changes in physical activity and physical function in DPSA users.

**Methods:**

We conducted a nonrandomized controlled trial of older adults aged ≥65 years. We recruited participants for 2 distinct 12-week programs designed to increase physical activity. Participants could choose between an intervention group (app program and exercise instruction) or a control group (exercise instruction only). DPSA creates a group chat for up to 5 people with a common goal, and participants anonymously post to each other in the group. Once a day, participants posted a set of their step counts, photos, and comments on a group chat box. The intervention group used the DPSA after receiving 2 face-to-face lectures on its use. The participants were characterized using questionnaires, accelerometers, and physical function assessments. The feasibility of the DPSA was assessed using retention and adherence rates. Physical activity was assessed using accelerometers to measure the daily step count, light intensity physical activity, moderate to vigorous intensity physical activity (MVPA), and sedentary behavior. Physical function was assessed using grip strength and the 30-second chair-stand test.

**Results:**

The participants in the intervention group were more frequent users of apps, were more familiar with information and communication technology, and had a higher baseline physical activity level. The retention and adherence rates for the DPSA intervention were 88% (36/41) and 87.7%, respectively, indicating good feasibility. Participants in the intervention group increased their step count by at least 1000 steps and their MVPA by at least 10 minutes using the DPSA. There was a significant difference in the interaction between groups and intervention time points in the daily step count and MVPA (step count, *P*=.04; duration of MVPA, *P*=.02). The DPSA increased physical activity, especially in older adults with low baseline physical activity levels.

**Conclusions:**

The feasibility of DPSA was found to be good, with the intervention group showing increases in daily steps and MVPA. The effects of DPSA on step count, physical activity, and physical function in older adults with low baseline physical activity should be investigated using randomized controlled trials.

## Introduction

### Background

The health benefits of regular physical activity are familiar [1]. Physical activity reduces the risk of chronic diseases such as type 2 diabetes, cardiovascular disease, and hypertension [2-4]. In addition, physical activity improves the overall physical and mental functioning and controls morbidity and mortality rates [5,6]. However, globally, the level of physical activity has remained stable or declined, despite several efforts to promote physical activity [7,8]. In addition, social distancing during the COVID-19 pandemic has caused changes in lifestyle and social behavior [9]. The level of physical activity among older adults in Japan is reported to have declined due to the COVID-19 pandemic [10,11] and needs to be increased.

Recently, mobile apps have been used successfully to increase physical activity levels [12,13]. eHealth encompasses health care services and information delivered with the aid of information and communication technology (ICT), including computers, mobile phones, and satellite communications. Mobile health (mHealth) refers to the use of smart or portable devices for providing health services and information [14]. With an increase in the population of older adults using the internet, mHealth and eHealth approaches may be feasible [15,16]. A total of 3 out of 4 reviews concluded that mHealth or eHealth interventions are effective in the short term in promoting physical activity in adults aged ≥50 years [17]. Furthermore, eHealth interventions targeting physical activity have revealed that theory-based interventions are more effective than interventions that are not grounded in theory [18]. However, there has been limited focus on social cognitive theory–based interventions aimed at promoting physical activity among older adults through peer support.

Peer support–based interventions have become a common method for promoting health-related behavior change [19]. Webel et al [20] defined peer support as “a method of teaching or facilitating health promotion that makes use of people sharing specific health messages with members of their own community.” The effectiveness of peer support–based interventions for physical activity has a theoretical basis, often explained by social cognitive theory [21]. The social cognitive theory proposed by Bandura [22] stipulates that behavior is learned by observing and imitating others. This process is called observational learning or modeling and has been extensively studied in the context of motor skill development and education. Peer-mediated delivery of information regarding physical activity through apps could facilitate attention, retention, and motivation to work on that information, as per social cognitive theory. Liu and Lachman [23] conducted a 4-week randomized controlled trial based on social cognitive theory in which older adults aged ≥60 years used the WeChat and WeRun apps to increase their step counts by recording and exchanging them through SMS text messages. This social engagement through SMS text messages increased the step count. However, the step count was the only physical activity outcome measure, and the effect of the intervention on physical activity intensity, sedentary behavior (SB), and physical function was not assessed. Therefore, using an app based on social cognitive theory, we examined the effects of a digital peer-supported intervention on step counts, physical activity intensity, SB, and physical function among older adults aged ≥65 years.

### Objective

This study used a digital peer-supported app (DPSA) to conduct a 12-week intervention study on older adults aged ≥65 years. The objectives of this study were threefold: (1) to understand the characteristics of older adults who choose to use the DPSA to increase their physical activity; (2) to evaluate the feasibility of using the DPSA to promote physical activity in older adults; and (3) to measure the effect of using the DPSA on users’ level of physical activity, SB, physical function, and self-efficacy for exercise.

## Methods

### Study Design

This nonrandomized pretest-posttest comparison trial of 2 groups was conducted in Fujisawa City, Kanagawa Prefecture, Japan. In April 2022, the city had an area of 69.57 km^2^ and a population of 442,892, of whom 108,472 (24.49%) were aged ≥65 years. The study was conducted as an industry-government-academia collaboration between the local government, an app-making company, and a university.

### Ethical Considerations

The study was approved by the Research Ethics Committee of Sports Medicine Research Center at Keio University (approval number 2022-07). Informed consent was obtained from all participants. The study protocol was registered with the University Hospital Medical Information Network (UMIN000050618).

### Participants

The study was conducted on Fujisawa City older adults aged ≥65 years. We recruited participants for 2 distinct 12-week programs designed to increase physical activity. Participants could choose between an intervention group (app program and exercise instruction) and a control group (exercise instruction only). Participants from different areas within the municipality were recruited through flyers, publicity, and calls to related organizations. The intervention was implemented in two phases: (1) from October 2022 to January 2023 and (2) from December 2022 to March 2023. Participants selected programs according to their preferences (the app program was for smartphone owners only). The eligibility criteria were older adults aged ≥65 years, who were able to walk independently and perform other activities of daily living and had not been advised to refrain from physical activity by a physician. Before participation, prospective participants were screened using a personal health status questionnaire based on the Physical Activity Readiness Questionnaire [24-26] to ascertain whether there were any potential problems with participation in the study. Because older adults are generally assumed to be less familiar with smartphones and apps than younger adults [27] and may not be able to completely use the DPSA, we provided 2 lectures about using the app; 1 lecture was conducted at the start of the DPSA use, and another was conducted 1 week later. Participants were instructed to download the app, and its use was explained during the first lecture. Each lecture lasted for 1 to 1.5 hours, and the participants were able to receive instructions directly from the instructor and ask questions.

### Intervention

#### Program

**Figure 1:**
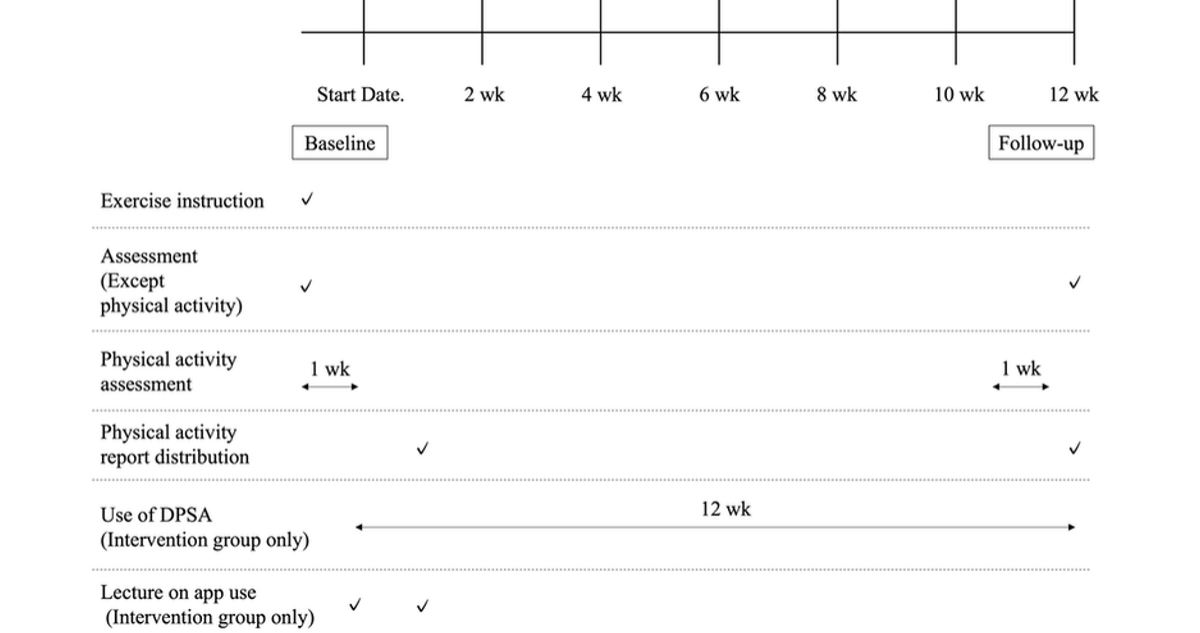
Timeline of study procedures. DSPA: digital peer support app; wk: weeks.

The timeline of the study procedure is presented in Figure 1. Regardless of program selection, all participants participated in face-to-face exercise instruction, program introduction, and baseline assessment conducted by a physical therapist or a health fitness instructor. Exercise instructions focused on aerobic, stretching, muscle strengthening, and balance exercises based on the original “Fujisawa +10 exercise” program [28,29]. Exercise instruction was provided for 15 minutes for the intervention group and 30 minutes for the control group. Both intervention and control groups were instructed to increase their daily physical activity. Participants completed questionnaires and underwent physical activity and physical function assessments using a triaxial accelerometer at baseline (start date) and during follow-up (weeks 10-12). Individual physical activity reports were generated from the data obtained and fed back to the participants. The intervention group began using the app 1 week after the baseline outcome assessment was conducted. The timing of the evaluation of each measurement item is described in Multimedia Appendix 1.

#### DPSA Intervention

**Figure 2:**
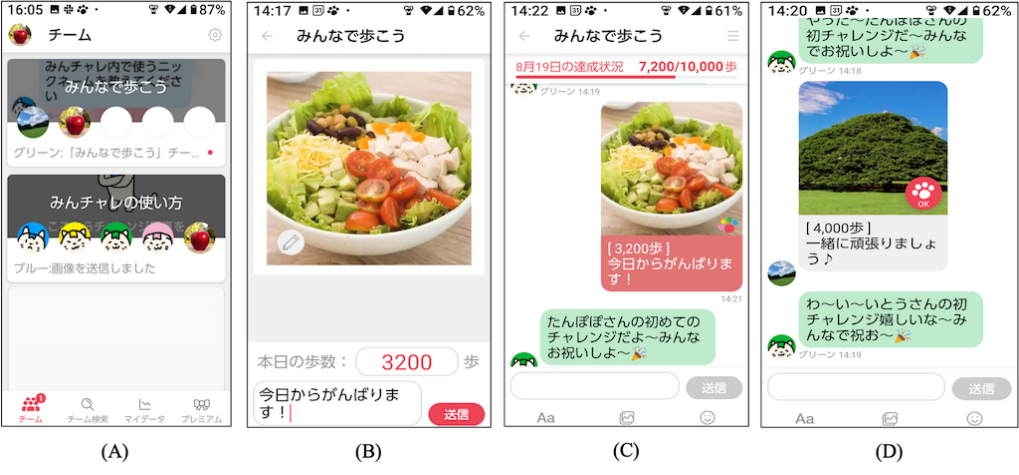
Examples of app screens. (A) Select a group. (B) Post photos, step count, and comments on the group. Post a photo taken that day and comment on the day’s events. (C) The contents of the posts are displayed in the group. The total number of steps for the group is displayed. (D) Response to posts by group members.

The study was conducted using Minchalle (A10 Lab Inc), a commercially available DPSA [30]. This app was developed in June 2015 and launched in November 2015. Figure 2 shows example screens from the app. The DPSA creates a group chat for up to 5 people with a common goal, and participants anonymously post messages to each other in the group. The common goal of the intervention group was to increase physical activity through walking and exercise. Once a day, participants posted a set of their step counts, photos, and comments on a group chat box. The main functions of the DPSA used in this study were to enable the participants to (1) post step counts, photos, and comments about the day; (2) post approvals from group members to each other’s postings; (3) set step count goals on a group basis; and (4) get feedback on the team’s total daily step count. Step counts were measured using a smartphone, with the DPSA reporting the number of steps taken on the day at the time of posting. Participants had the option to post comments or photos multiple times a day and engage with other members, although this was not mandatory. The app was available for participants to use free of charge.

### Measurements

#### Demographic Characteristics of Participants

In addition to general characteristics such as age and sex, the survey asked about smartphone ownership, the frequency of app use, exercise habits, the frequency of neighborhood interaction, participation in group exercise, history of falls in the past year, the effect of the COVID-19 pandemic on their level of physical activity, self-reported decrease in walking speed, the use of ICT, and self-efficacy for exercise.

Participant’s body weight (kg) was measured using a digital scale, and height (m) was measured on a stadiometer after participants removed their shoes. BMI was then calculated as body weight divided by the square of height.

Exercise habits were defined as “those who exercise at least twice a week, for at least 30 minutes each time, for at least one year” [31].

The frequency of neighborhood interaction was assessed by asking how many times one interacts with people in the neighborhood within 1 week.

Group exercise participation was defined as those who participate in a group of three or more people who meet voluntarily to exercise.

Information about the use of ICT was collected using questions about “Gathering information and shopping on the internet,” “Using social networking services (Facebook, LINE, Instagram, etc),” and “Do not use any information devices.” The percentages for the intervention and control groups were compared with representative values from the Annual Report on the Ageing Society, published by the Japanese Cabinet Office, to determine the extent to which participants are using ICT compared with other older adults [32].

#### Outcome Measures of Participants

To assess physical activity, participants were asked to wear a triaxial accelerometer [33] (Active Style Pro HJA-750C Activity Meter, Omron Health Care) at waist level for 7 consecutive days before the intervention and 10 to 11 weeks after the intervention commenced. The accelerometer display was configured to prevent users from viewing the amount of physical activity for the day. Participants were instructed not to remove the device unless required for certain tasks, such as changing their clothes and bathing. At the end of the measurement, all the data collected were transferred from the accelerometer to a PC. Following the method suggested by Jefferis et al [34] for estimating physical activity, an individual needed to record at least 10 hours of activity per day for 3 days to be included in the subsequent analyses. The data were collected in 60-second epochs for data analysis and used to estimate the intensity of activity in metabolic equivalents (METs). The mean daily step count and time spent in SB (≤1.5 METs), light intensity physical activity (LPA; 1.6-2.9 METs), and moderate to vigorous intensity physical activity (MVPA: ≥3 METs) per day were used for outcome measurements of physical activity.

Physical function was assessed using grip strength and the 30-second chair-stand test (CS-30). Grip strength was measured using a digital dynamometer (Grip D, TKK 5401, Takei Scientific Instruments). Measurements were taken in the standing position, with the elbow joint in extension and the wrist joint in midextension. Both the left and right hands were measured once each, and the maximum value was used. For the CS-30 test [35], seated participants were instructed to stand up from the chair with their arms crossed at chest level as many times as possible in 30 seconds.

Self-efficacy for exercise was assessed using 4 questions pertaining to participants’ self-confidence in exercising under each of the following conditions [36]: “Do you have the confidence to exercise regularly under the following conditions? physical fatigue, mental stress, lack of time, and bad weather.” In response to the question, participants were asked to select 1 of the 5 answers ranging from “No, I don’t have any confidence at all (1 point)” to “Yes, I am quite confident (5 points).” The total score ranged from 4 to 20. Textbox 1 summarizes the measures related to the characteristics of the participants.

**Measurements related to participant characteristics.**

**Measurement methods and items**

- Questionnaire
  Age, sex, living alone, self-rated health, perceived household economic status, life satisfaction, employment status, smartphone ownership, frequency of app use, exercise habits, frequency of neighborhood interaction, participation in group exercise, history of falls in the past year, effect of the COVID-19 pandemic on decreased physical activity, self-reported decrease in walking speed, and self-efficacy for exercise
- Triaxial accelerometer
  Steps, light intensity physical activity, moderate to vigorous intensity physical activity, and sedentary behavior
- Physical function assessment
  BMI, grip strength, and 30-second chair-stand test

#### Feasibility of DPSA Intervention

The feasibility of DPSA intervention was assessed by retention and adherence rates during the 12-week program implementation. The DPSA could exclude a person from a group if they have not posted a set of their step counts, photos, and comments for 15 consecutive days. Dropouts were defined as those who were excluded from the group during the 12 weeks of DPSA intervention. The retention rate was calculated using a denominator of 41 participants including those who withdrew consent. The adherence rate of DPSA intervention was calculated by dividing the number of sets of their step counts, photos, and comments posted during the intervention period by the duration of the intervention. DPSA adherence rates were also calculated by group (9 groups: A-I). The number of all chat posts per person by group was calculated to assess group use. Negative physical effects that occurred during the intervention were ascertained by interviewing the participants during follow-up. We report on privacy breaches and technical problems with the app. Privacy breaches were identified by the municipality, and technical problems were identified by the app company. Continuity was evaluated using a questionnaire on factors that contributed to exercise continuation by the DPSA and the intention to continue using the DPSA after 12 weeks.

#### Changes in Physical Activity, SB, Physical Function, and Self-Efficacy for Exercise

Physical activity (step count, LPA, MVPA, and SB); physical function (grip strength and CS-30); and self-efficacy for exercise were assessed at 2 time points: baseline and follow-up. The follow-up data were measured in the same way as at baseline. For follow-up data, physical activity was measured between weeks 10 and 11 of the intervention. Physical function and self-efficacy for exercise were measured after 12 weeks of the intervention.

### Statistical Analysis

Comparisons of participant characteristics in each group were analyzed using the independent samples *t* test (2-tailed), chi-square test, and Mann-Whitney *U* test. The interaction between the group and time of intervention was analyzed using a linear mixed model with baseline and follow-up group differences adjusted for baseline age, sex, and frequency of app use (at baseline). Consent withdrawers were excluded, and older adults who dropped out of the intervention were included in the analysis in a modified intention-to-treat analysis. The daily step count, SB, LPA, MVPA, grip strength, CS-30, and self-efficacy for exercise were analyzed as dependent variables in separate models. The daily step counts were positively skewed; therefore, square root transformations were applied to improve normality. To increase the comprehensibility of the tables, raw descriptive data were reported, although analyses were conducted using the square root–transformed values. We defined high and low levels of physical activity based on step counts, using 7000 steps per day as the cutoff, as the recommended step goal for older adults is typically 7000 to 10,000 steps [37]. To examine the impact of physical activity at baseline on the intervention effect, a post hoc subgroup analysis was conducted on participants in the intervention group who had different levels of physical activity at baseline (≥7000 steps per day vs <7000 steps/day). The interaction between the physical activity level and intervention time point was analyzed in a linear mixed model adjusted for baseline age, sex, and frequency of app use. The data were analyzed using SPSS (version 29.0; IBM Corp). The statistical significance level was set at 5%.

## Results

### Participants

**Figure 3:**
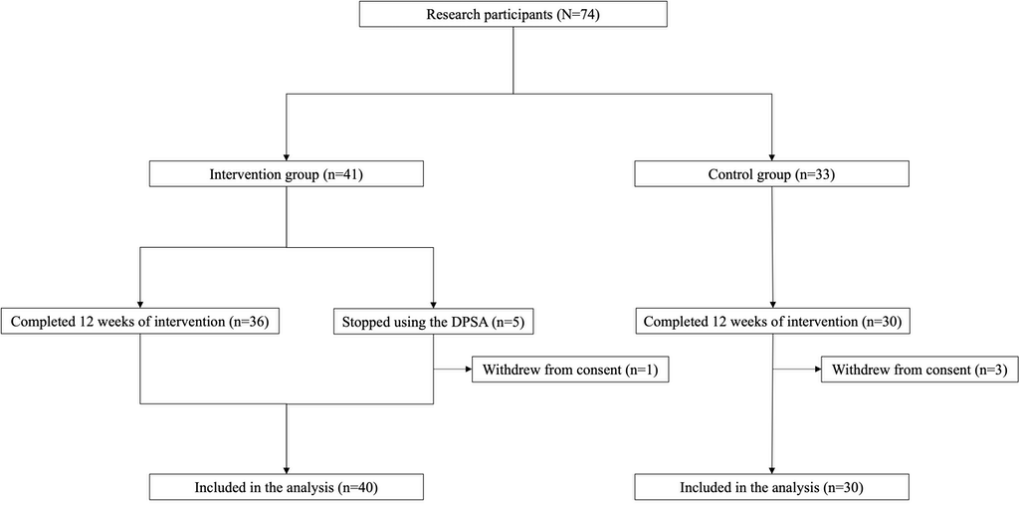
Flow diagram of participants. DPSA: digital peer support app.

A total of 74 participants were initially enrolled in the study. However, 4 (5%) withdrew consent during the 12-week intervention period, with 1 (25%) withdrawal in the intervention group and 3 (75%) in the control group. The final analysis included 70 participants, 40 (57%) in the intervention group and 30 (43%) in the control group, in a modified intention-to-treat analysis, excluding those who withdrew consent (Figure 3). The intervention group comprised 9 groups of 4 to 5 participants each. A total of 5 (12%) out of 41 participants in the intervention group dropped out, including the 1 who withdrew consent.

### Participant Characteristics

The baseline participant characteristics are shown in Tables 1 and 2. The mean age of the participants (n=70) was 77.3 (SD 6.1) years, with 26 (37%) male participants included in the study. There were no differences in baseline demographic characteristics between the intervention and control groups. However, participants in the intervention group were more likely to use apps and exercise more frequently. “Gather information and shop on the internet” for the intervention group, the control group and representative percentage were 78%, 57%, and 23.7%, respectively. “Use social networking services” for the intervention group, the control group and representative percentage were 60%, 37%, and 13.1%, respectively. “Do not use any information devices” for the intervention group, the control group and representative percentage were 0%, 3%, and 17%, respectively. Compared to the representative percentage of Japanese older adults based on the Annual Report on the Ageing Society published by the Japan Cabinet Office [32], both groups used ICT, with the intervention group exhibiting greater ICT use compared to the control group.

Although the difference in the baseline daily step count between groups was not statistically significant, the step count was higher in the intervention group than in the control group, with a median difference of >1000 steps. There was a statistically significant difference in baseline LPA and MVPA between the 2 groups; the intervention group exhibited significantly higher MVPA levels, while the control group showed significantly higher LPA levels. Furthermore, grip strength was higher in the intervention group, likely owing to the greater proportion of male participants; however, the difference was not statistically significant.

**Table 1:** Demographic characteristics of the participants (n=70).

Characteristics			Total sample		Intervention group (n=40)		Control group (n=30)		*P* value	
Age (years), mean (SD)			77.3 (6.1)		76.9 (6.1)		77.9 (6.1)		.49^a^	
**Sex, n (%)**										.12^b^
	Male	26 (37)		18 (45)		8 (27)				
	Female	44 (63)		22 (55)		22 (73)				
BMI (kg/m^2^), mean (SD)			22.8 (2.9)		23.0 (2.9)		22.4 (2.9)		.43^a^	
Living alone, n (%)			19 (27)		13 (32)		6 (20)		.22^b^	
**Self-rated health, n (%)**										.13^b^
	Excellent, good, or normal	63 (90)		38 (95)		25 (83)				
	Fair or poor	7 (10)		2 (5)		5 (17)				
**Perceived household economic status, n (%)**										.57^b^
	Excellent, good, or normal	67 (96)		39 (98)		28 (93)				
	Fair or poor	3 (4)		1 (2)		2 (7)				
**Life satisfaction, n (%)**										.53^b^
	Excellent, good or normal	62 (89)		35 (88)		27 (90)				
	Fair or poor	8 (11)		5 (12)		3 (10)				
Working, n (%)			18 (26)		9 (22)		9 (30)		.48^b^	
Smartphone ownership, n (%)			67 (96)		40 (100)		27 (90)		.07^b^	
**Frequency of app use, n (%)**										.07^b^
	Usually or sometimes	54 (77)		34 (85)		20 (67)				
	Rarely or never	16 (23)		6 (15)		10 (33)				
Exercise habits, n (%)			37 (53)		24 (60)		13 (43)		.17^b^	
**Frequency of neighborhood interaction, n (%)**										.53^b^
	≥3 times/week	31 (44)		19 (48)		12 (40)				
	≤2 times/week	39 (56)		21 (52)		18 (60)				
Participation in group exercise, n (%)			34 (49)		18 (45)		16 (53)		.49^b^	
History falls in the past year, n (%)			9 (13)		5 (12)		4 (13)		.60^b^	
**Effect of the COVID-19 pandemic on decreased physical activity, n (%)**										.64^b^
	Great or slight	51 (73)		30 (75)		21 (70)				
	Not much or unchanged	19 (27)		10 (25)		9 (30)				
Self-reported decrease in walking speed, n (%)			49 (70)		28 (70)		21 (70)		>.99^b^	

^a^Analysis was conducted using the independent samples *t* test (2-tailed).

^b^Analysis was conducted using the chi-square test.

**Table 2:** Baseline measures of the triaxial accelerometer, physical function assessment, and self-efficacy for exercise (n=70).

Outcome measures		Total sample	Intervention group (n=40)	Control group (n=30)	*P* value
**Triaxial accelerometer^a^**					
	Steps/day, median (IQR)	5511 (3783-7852)	6310 (3936-8132)	5276 (3522-6275)	.08^b^
	LPA^c^ (minutes/day), mean (SD)	329.9 (91.0)	306.6 (79.7)	360.3 (97.0)	.01^d^
	MVPA^e^ (minutes/day), mean (SD)	45.5 (26.5)	51.0 (24.1)	38.4 (28.1)	.049^d^
	SB^f^ (minutes/day), mean (SD)	538.2 (110.3)	530.1 (86.0)	522.3 (116.8)	.54^d^
	Triaxial accelerometer wearing time (minutes/day), mean (SD)	913.6 (121.8)	888.4 (80.0)	946.4 (159.6)	.07^d^
**Physical function**					
	Grip strength (kg), mean (SD)	25.6 (8.1)	26.9 (8.7)	23.8 (7.0)	.12^d^
	CS-30^g^, mean (SD)	20.7 (6.4)	20.7 (7.2)	20.8 (5.2)	.92^d^
Self-efficacy for exercise, mean (SD)		13.6 (3.6)	13.9 (3.6)	13.3 (3.6)	.54^d^

^a^Participants with valid accelerometer data; total sample, n=69; intervention, n=39; and control, n=30.

^b^Analysis was conducted using the Mann-Whitney *U* test.

^c^LPA: light intensity physical activity.

^d^Analysis was conducted using the independent samples *t* test (2-tailed).

^e^MVPA: moderate to vigorous intensity physical activity.

^f^SB: sedentary behavior.

^g^CS-30: 30-second chair-stand test.

### Feasibility: Retention Rate, Number of Posts, Negative Impact, Continuation Factors, and Willingness to Continue

A total of 5 (12%) out of the 41 participants in the intervention group dropped out, resulting in a DPSA continuation rate of 88% (36/41). Reasons for dropping out included “withdrew research consent,” “not a good fit for me,” and “unknown cause” (n=1, 20% each), as well as “poor health” (n=2, 40%). The average number of total posts per person was 2.76 (SD 1.99) per day. After excluding participants who dropped out, the adherence rate was 96%, and the average number of total posts per person was 3.02 (SD 1.93) per day (Table 3).

**Table 3:** Digital peer support app adherence rate and average number of total posts per day among participants in the intervention group.

Group	All participants (n=40)			Excluding participants who drop out (n=36)	
	n (adherence rate, %)	Total posts/person/day, mean (SD)	n (adherence rate, %)		Total posts/person/day, mean (SD)
All	40 (87.7)	2.76 (1.99)	36 (95.9)		3.02 (1.93)
A	4 (95.8)	1.55 (0.32)	4 (95.8)		1.55 (0.32)
B	5 (88.3)	1.83 (0.88)	5 (88.3)		1.83 (0.88)
C	5 (80.4)	2.62 (1.72)	4 (98.6)		3.24 (1.15)
D	5 (79.8)	1.12 (0.59)	4 (99.2)		1.38 (0.15)
E	5 (77.1)	1.19 (1.72)	4 (91.6)		1.22 (0.32)
F	4 (99.7)	6.25 (1.46)	4 (99.7)		6.25 (1.46)
G	4 (98.6)	3.36 (1.16)	4 (98.6)		3.36 (1.16)
H	4 (95.5)	4.20 (1.07)	4 (95.5)		4.20 (1.07)
I	4 (74.1)	3.83 (2.88)	3 (96.2)		4.88 (2.40)

**Figure 4:**
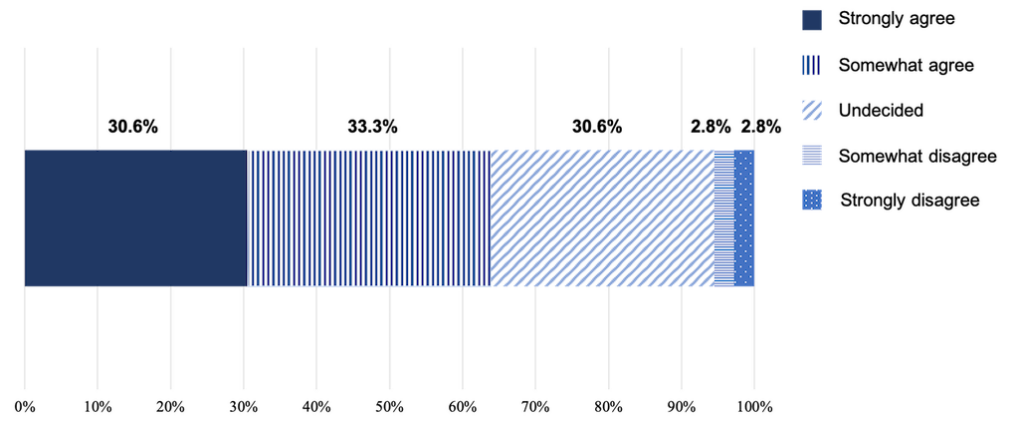
Intention to continue using the digital peer support app after the end of the study among participants in the intervention group.

A total of 3 minor negative physical effects were reported; 2 participants reported knee pain and 1 reported plantar pain. There were no breaches of privacy associated with the use of the app. A total of 14 inquiries were received about how to use the app. The average response took approximately 15.4 (10.7) minutes per case. The reported reasons for continuing to use the app were continuing fellowship (28/36, 78%), having a common goal (24/36, 67%), having fun (20/36, 56%), tracking their step counts (20/36, 56%), maintaining motivation (18/36, 50%), sense of improved mental health (17/36, 47%), sense of improved physical health (14/36, 39%), being on the internet (14/36, 39%) and Emotional painless (11/36, 31%). Figure 4 presents the intention to continue using the DPSA after the 12-week intervention. The responses were strongly agree (11/36, 31%), somewhat agree (12/36, 33%), undecided (11/36, 31%), somewhat disagree (1/36, 3%), and strongly disagree (1/36, 3%). More than half of the participants (23/36, 64%) indicated an intention to continue.

### Changes in Physical Activity, Physical Function, and Self-Efficacy for Exercise

The results of the linear mixed model analysis of physical activity, physical function, and self-efficacy for exercise are listed in Table 4. There was a significant difference in the interaction between groups and intervention time points in the daily step count and MVPA (daily step count: *P*=.04 and MVPA: *P*=.03) but not in LPA, SB, grip strength, CS-30, and self-efficacy for exercise.

A post hoc subgroup analysis was conducted by dividing the intervention group into high-level physical activity and low-level physical activity subgroups [37] based on their level of physical activity at baseline. The step count, LPA, MVPA, and SB were compared (Table 5). The analysis showed a significant difference in the interaction between the groups and the daily step count at baseline (*P*=.04).

**Table 4:** Intervention effects on physical activity, physical function, and self-efficacy for exercise before and after the intervention.

Outcome measures^a^			Intervention group					Control group					Group×time (adjusted) *P* value
			Baseline		Follow-up		Baseline			Follow-up			
**Accelerometer data^b^**													
	Steps/day, median (IQR)	6310 (3936-8132)		8368 (5331-10,235)		5276 (3522-6275)			5143 (2715-6648)		.04		
	LPA^c^ (minutes/day), mean (SD)	306.6 (79.7)		303.6 (91.0)		360.3 (97.0)			332.2 (81.7)		.06		
	MVPA^d^ (minutes/day), mean (SD)	51.0 (24.1)		65.7 (32.0)		38.4 (28.1)			38.4 (28.1)		.02		
	SB^e^ (minutes/day), mean (SD)	530.1 (86.0)		522.3 (116.8)		522.3 (116.8)			549.4 (113.7)		.55		
Grip strength^f^ (kg), mean (SD)			26.9 (8.7)		26.0 (9.3)		23.8 (7.0)			24.6 (7.4)		.09	
CS-30^g^, mean (SD)			20.7 (7.2)		22.2 (7.3)		20.8 (5.3)			20.4 (6.3)		.50	
Self-efficacy for exercise^h^, mean (SD)			13.9 (3.6)		14.8 (3.1)		13.3 (3.7)			14.0 (3.6)		.70	

^a^Analyses were adjusted for age, sex, and frequency of app use (at baseline).

^b^Participants with valid accelerometer data; intervention group: baseline, n=39, and follow-up, n=35; control group: baseline, n=30, and follow-up, n=30.

^c^LPA: light intensity physical activity.

^d^MVPA: moderate to vigorous intensity physical activity.

^e^SB: sedentary behavior.

^f^Participants with valid grip strength data; intervention group: baseline, n=40, and follow-up, n=34; control group: baseline, n=30, and follow-up, n=23.

^g^CS-30; 30-second chair-stand test; participants with valid data; intervention group: baseline, n=40, and follow-up, n=32; control group: baseline, n=30, and follow-up, n=22.

^h^Participants with valid self-efficacy for exercise data; intervention group: baseline, n=40, and follow-up, n=36; control group: baseline, n=30, and follow-up, n=28.

**Table 5:** Comparison of intervention effects on accelerometer data between low-level and high-level physical activity subgroups at baseline and follow-up in the intervention group.

Outcome measures^a^ (accelerometer data)^b^	<7000 steps/day (n=22)		≥7000 steps/day (n=18)		Group×time (adjusted) *P* value
	Baseline	Follow-up	Baseline	Follow-up	
Steps/day, median (IQR)	4338 (3207-5495)	5761 (4649-8680)	8581 (7571-10,117)	9277 (8133-10,980)	.007
LPA^c^ (minutes/day), mean (SD)	296.7 (78.1)	301.1 (94.8)	318.0 (82.3)	306.2 (89.5)	.25
MVPA^d^ (minutes/day), mean (SD)	33.1 (12.7)	52.0 (31.0)	71.9 (15.7)	80.2 (26.7)	.18
SB^e^ (minutes/day), mean (SD)	538.2 (92.1)	513.3 (126.1)	522.4 (80.0)	531.7 (109.1)	.15

^a^Analyses were adjusted for age, sex, and frequency of app use (at baseline).

^b^Participants with valid accelerometer data; low-level physical activity (<7000 steps/day) group: baseline, n=21, and follow-up, n=18; high-level physical activity (≥7000 steps/day) group: baseline, n=18, and follow-up (n=18).

^c^LPA: light intensity physical activity.

^d^MVPA: moderate to vigorous intensity physical activity.

^e^SB: sedentary behavior.

## Discussion

### Principal Findings

The study aimed to determine the characteristics of older adults who wanted to use the DPSA, which aimed to increase physical activity among older adults, and to confirm the feasibility of the DPSA and its impact on physical activity. Older adults who wanted to use the DPSA were more likely to be frequent users of the app and were more familiar with the use of ICT. Participants who reported an exercise habit tended to be more physically active at baseline. The retention rate was 88% (36/41) and the adherence rate was 87.7%, demonstrating the feasibility of older adults using the DPSA. The step count and MVPA increased significantly in the intervention group compared with those in the control group, demonstrating that the DPSA effectively increased physical activity. In DPSA users, participants with lower levels of baseline physical activity showed a more significant increase in their daily step count compared with those with higher levels of physical activity.

### Comparison With Previous Studies

In this study, the retention and adherence rates were 88% (36/41) and 87.7%, respectively. These values are favorable compared to those reported in previous studies that have used digital technology to increase physical activity among older adults [38-42]. The findings from this study show that it is feasible for older adults to use DPSA to increase their level of physical activity. While differences were observed in adherence rates and the average number of total posts per day between groups, it is unclear what factors contribute to these differences. Only 3 negative physical effects were reported, but they were all minor and did not cause privacy breach issues. In contrast to our findings, Kullgren et al [43] showed that peer support using a 4-person web-based SMS text message board did not lead to an increase in physical activity among older adults. The authors attributed this lack of effectiveness to the failure to facilitate communication. In this study, the average number of comments per day in the intervention group was 2.76 overall and 3.01 excluding dropouts, indicating that many participants were actively using the DPSA. In addition, the fellowship was the factor with the highest percentage of intention to continue using DPSA. These may indicate that peer support based on social cognitive theory increased physical activity, as hypothesized. Self-efficacy is a key aspect of social cognitive theory [21]. However, in this study, although there was an increase in self-efficacy for exercise scores, the change was not significant. Possible reasons for the lack of a significant increase in self-efficacy for exercise in this study include the high baseline self-efficacy for exercise of the study population, the ceiling effect, and the short intervention period. In addition, the questionnaire used in this study may not reflect the impact of DPSA on self-efficacy. In this study, DPSA users showed a significant increase in their daily step count and MVPA duration despite the winter season. Participants with lower baseline physical activity levels showed a greater increase than those with higher levels of physical activity, suggesting that older adults with lower levels of physical activity may benefit more from using the DPSA than those with higher levels of physical activity. In the intervention group, the daily step count increased by >1000 steps on average. A systematic review of 17 prospective studies by Hall et al [44] has shown that each 1000-step increase in the daily step count decreases the risk of death and heart disease, with a 6% to 36% decrease in all-cause mortality risk and a 5% to 21% decrease in heart disease risk. Furthermore, it has been shown that an increase of 1000 steps per day decreases a woman’s risk of diabetes by 6% and an increase of 2000 steps per day decreases the risk of diabetes by 12% [45]. In this study, the duration of MVPA increased by >10 minutes. Previous studies conducted in the United States [46] have shown that adding 10 minutes per day of MVPA could prevent 6.9% of deaths per year in the US adult population aged between 45 and 85 years. A greater increase in physical activity is predicted to have a greater protective effect. In Japan, the Ministry of Health, Labour and Welfare published the ActiveGuide, the Japanese official physical activity guidelines for health promotion, in March 2013 [47]. The key message of this guideline is “+10,” indicating “add 10 minutes of MVPA per day.” We increased physical activity in older adults through a 5-year community-wide intervention that incorporated this guideline [48]. According to a meta-analysis of 26 cohort studies by Miyachi et al [49], an increase of 10 minutes of MVPA per day can cause a 3.2% reduction of the average relative risk of noncommunicable diseases, dementia, joint-musculoskeletal impairment, and mortality. The 2010 National Health and Nutrition Survey [50] found that 60.8% of the respondents are willing to take part in an additional 10 minutes of physical activity per day. Therefore, the “+10” recommendation could be feasible and efficient for the Japanese population [49]. On the basis of previous findings and the results of this study, DPSA is a viable and effective tool for increasing physical activity.

### Limitations

This study has a few limitations. First, sampling problems such as small sample size and low statistical power, as well as the influence of confounding factors such as academic background, digital literacy, and motivation, cannot be ruled out. Indeed, there was a difference between the 2 groups regarding the percentage of information about the use of ICT. The enrolment to the intervention and control groups was nonrandomized, and participation was voluntary. A previous study by Tudor-Locke et al [37] has shown that healthy older adults tend to walk between 2000 and 9000 steps per day, with a median of 5500 steps. The control group had about the same number of steps as the average older adult in Japan, whereas the intervention group took approximately 1000 more steps per day than the average older adult in Japan. Both groups were highly interested in exercise, which might have influenced the effectiveness of the DPSA intervention. Second, this study used a short-term intervention period of 12 weeks. Previous studies have found that mHealth physical activity interventions are more effective over short periods of time (<16 and <12 weeks) than over longer periods of time and that the effects may not be maintained for longer periods of time [51,52]. Furthermore, other reviews have shown that mHealth interventions may promote small to moderate increases in physical activity and that the effects are maintained over time but that the effect size decreases over time [53]. In this study, 6% (2/36) of the participants stated that they did not have intention to continue the intervention after 12 weeks and 31% (11/36) answered undecided, indicating that high retention and adherence rates can be achieved for short-term use of 12 weeks but that the long-term retention rate is unknown. Third, the generalizability is limited, as participation in the intervention group was limited to those who owned a smartphone. In addition, they were already familiar with the use of ICT. In this study, training sessions were provided on the use of the app, so that even those who were less familiar with the use of the app could participate, but participation was low.

### Conclusions

In this study, a 12-week intervention was conducted with older adults aged ≥65 years, using DPSA to increase physical activity. Older adults who used DPSA to increase physical activity tended to be more familiar with ICT and more physically active at baseline compared to the general older adult population. The feasibility of DPSA was good, with increases in daily steps and MVPA in the intervention group. Peer support–based interventions using digital apps may be effective in promoting physical activity among older adults. Notably, participants with lower levels of baseline physical activity showed a more significant increase in their daily step count compared with those with higher levels of physical activity. To confirm the effect of DPSA on physical activity and physical function in older adults, a randomized controlled trial should be conducted.

## Appendix

Multimedia Appendix 1 The timing of the evaluation of each measurement item.

## Abbreviations

CS-30: 30-second chair-stand test
DPSA: digital peer support app
ICT: information and communication technology
LPA: light intensity physical activity
MET: metabolic equivalent
mHealth: mobile health
MVPA: moderate to vigorous intensity physical activity
SB: sedentary behavior
