# Supplementary material for: Digital Peer-Supported App Intervention to Promote Physical Activity Among Community-Dwelling Older Adults: Nonrandomized Controlled Trial
Source: JMIR Aging. 2024 May 30;7:e56184. doi: 10.2196/56184 (PMC11176879; doi:10.2196/56184)
Supplement: Multimedia Appendix 1 [file aging_v7i1e56184_app1.docx]

**Multimedia Appendix 1.** The timing of the evaluation of each measurement item.

| **Time of measurement items** | | | |
| --- | --- | --- | --- |
|  | Baseline | Intervention period (12 weeks) | Follow-up |
| **Participants characteristics** |  |  |  |
| General characteristics | ✓ |  | ✓ |
| Smartphone owner | ✓ |  | ✓ |
| Frequency of app use | ✓ |  | ✓ |
| Exercise habits | ✓ |  | ✓ |
| Frequency of neighborhood interactions | ✓ |  | ✓ |
| Participation in group exercise | ✓ |  | ✓ |
| History of falls in the past year | ✓ |  | ✓ |
| Effect of COVID-19 on decreased physical activity | ✓ |  | ✓ |
| Self-reported decrease in walking speed | ✓ |  | ✓ |
| **Outcomes** |  |  |  |
| Physical activity (Steps, LPA, MVPA, SB)^a^ | ✓ | ✓ |  |
| Grip strength | ✓ |  | ✓ |
| CS-30 | ✓ |  | ✓ |
| Self-efficacy for exercise | ✓ |  | ✓ |

a: For seven consecutive days before the intervention and 10 to 11 weeks after the intervention
